# Visual Field Indices or Retinal Nerve Fiber Layer? Predictors of Relative Afferent Pupillary Defect Severity in Asymmetric Glaucoma

**DOI:** 10.12669/pjms.42.8.14773

**Published:** 2026-08

**Authors:** Yousaf Jamal Mahsood, Alina Raza Khan, Anam Khan, Hafsah Aman, Saima Farooq, Rashid Zia

**Affiliations:** 1 Yousaf Jamal Mahsood, MBBS, MHR, FICO, FRCS, FCPS. Associate Professor, Department of Ophthalmology, Khyber Girls Medical College, Hayatabad Medical Complex, Peshawar, Pakistan; 2 Alina Raza Khan, MBBS. Trainee Medical Officer, Department of Ophthalmology, Khyber Girls Medical College, Hayatabad Medical Complex, Peshawar, Pakistan; 3 Anam Khan, MBBS. Trainee Medical Officer, Department of Ophthalmology, Khyber Girls Medical College, Hayatabad Medical Complex, Peshawar, Pakistan; 4 Hafsah Aman, MBBS. Trainee Medical Officer, Department of Ophthalmology, Khyber Girls Medical College, Hayatabad Medical Complex, Peshawar, Pakistan; 5 Saima Farooq, MBBS, FCPS. Assistant Professor, Department of Ophthalmology, Khyber Girls Medical College, Hayatabad Medical Complex, Peshawar, Pakistan; 6 Rashid Zia, MBBS, MRCSEd. Consultant Ophthalmologist, East Kent Hospitals University NHS Foundation Trust, Ashford, GB, United Kingdom

**Keywords:** Afferent Pupillary Defect, Glaucoma, Optical Coherence Tomography, Retinal Nerve Fiber Layer, Visual Fields

## Abstract

**Objective:**

To determine the association of visual field indices like mean deviation (MD) and visual field index (VFI) and retinal nerve fiber layer (RNFL) thickness with relative afferent pupillary defect (RAPD) in asymmetric glaucoma and to quantify the amount of inter-eye differences in these parameters required to predict severity of RAPD grades.

**Methodology:**

This study was conducted at glaucoma clinic of ophthalmology department of Hayatabad Medical Complex, Peshawar, Pakistan from July 2024 to March 2025. Patients diagnosed with asymmetric glaucoma (all types) and clinically detectable RAPD were included in this study. RAPD was recorded using clinical plus grading. The RNFL thickness, mean deviation (MD), visual field index (VFI), and their inter-eye differences were calculated. Ordinal logistic regression assessed the relationship between RAPD grades and the inter-eye differences in these parameters. A p-value<0.05 was set for statistical significance.

**Results:**

In 126 participants recruited, the mean age was 52.48 ± 14.63 years and 97 (77%) were males. The inter-eye differences in OCT-RNFL (global or sectoral), MD and VFI were statistically significant (p<0.001). Inter-eye difference in MD was the only significant predictor of RAPD grade (OR=1.225, 95% CI=1.009-1.489, p=0.04), suggesting that greater differences in MD are associated with higher RAPD severity. The association between the glaucoma stage of the worse eye and RAPD grades were found statistically insignificant (p=0.345).

**Conclusions:**

Inter-eye differences in RNFL thickness, MD, and VFI were significantly associated with RAPD severity. Visual field asymmetry (MD difference) emerged as the strongest predictor of RAPD grade, while glaucoma stage showed no association with RAPD severity.

## INTRODUCTION

Relative afferent pupillary defect (RAPD) is an important clinical sign for identifying and measuring neuronal dysfunction in conditions affecting the retina and optic nerve.^1^ It usually appears in unilateral or asymmetric anterior visual pathway abnormalities, of which glaucoma is a well-known cause.^2^,^3^ The hallmark of glaucomatous optic neuropathy is the gradual loss of retinal ganglion cells, which eventually results in impairment of the visual field. One Study also suggests that there is loss of macular volume in glaucoma patients as compared to normal.^4^

Although glaucoma is usually bilateral, asymmetric involvement is also observed, that results in inter-eye variations in retinal nerve fiber layer (RNFL) thickness and standard automated perimetry (SAP) sensitivity.^5^ It requires a considerable amount of asymmetric neuronal damage for RAPD to become clinically evident.^6^ The assessment of pupillary light reflex in glaucoma has been thoroughly investigated, and a number of techniques have been put forth for identifying and grading RAPD, such as clinical plus grading, the use of neutral density filters, and virtual reality (VR) headsets.^7^-^9^ Among these, clinical plus grading remains a practical and widely used approach in many clinical settings due to its simplicity, rapid applicability, and minimal equipment requirements. This method is often integrated into routine ophthalmic examinations, even in centers that have access to advanced technologies like optical coherence tomography (OCT) and Humphrey field analyzer (HFA). Additionally, prior studies have shown that clinical plus grading provides results comparable to more equipment-intensive methods, supporting its continued relevance in both research and practice.^10^,^11^

In patients with glaucoma, RNFL thickness is frequently measured using OCT to assess structural damage.^12^ According to Tatsumi et al., when the RNFL thickness in the affected eye drops to around 75% of that in a healthy eye, RAPD becomes clinically detectable;^1^ however, their study did not measure or correlate the Visual Field Index (VFI)—an important parameter of visual function—with RAPD in asymmetric glaucoma. On the other hand, a Thai study discovered a considerable correlation between RAPD and visual field indices, notably the Visual Field Index (VFI) and Mean Deviation (MD), with VFI demonstrating the strongest association.^10^ However, the study’s small sample size, potential interobserver variability in RAPD grading, and heterogeneity of clinical diagnoses limit the validity and applicability of its findings in asymmetric glaucoma.

Additionally, while it is known that RAPD severity in glaucoma correlates most strongly with visual field loss within 30 degrees eccentricity,^13^ it remains unclear whether VFI or MD serves as a better predictor of RAPD severity. Considering that both indices are influenced by central visual field defects, this uncertainty highlights the necessity for a more thorough evaluation. Further complicating the current understanding, Mishra et al. recommended disease-specific investigations to accurately define the relationship between RAPD and RNFL thinning.^2^ This highlights the necessity of condition-specific research that is solely focused on glaucoma to provide more precise understanding. The study focuses on analyzing RAPD in glaucoma patients and its correlation with RNFL thickness and visual field characteristics.

It investigates whether differences in visual field indices and RNFL thickness between eyes relate to the presence and severity of RAPD. By concentrating solely on asymmetric glaucoma, the research aims to provide a clearer understanding of the structural and functional damage associated with RAPD. Utilizing a standard clinical scale for RAPD grading, the findings may offer ophthalmologists a practical approach to assess glaucomatous damage when specialized equipment is unavailable.

## METHODOLOGY

This was an observational study conducted at glaucoma clinic of ophthalmology department of Hayatabad Medical Complex, Peshawar, Pakistan from July 2024 to March 2025.

### Ethical approval:

The study was granted ethical approval (IREB No. 1973; date July 1, 2024) and adhered to the standards of declaration of Helsinki.

### Inclusion criteria:

Patients diagnosed with all types of glaucoma who aged between 15 to 75 years and presented with a clinically detectable RAPD were included in this study.

### Exclusion criteria:

Those having history of ocular trauma, uveitis, pupillary abnormalities other than RAPD, asymmetric cataracts, any retinal pathology, refractive error of worse than +3 diopter or -6 diopter of spherical equivalent (SE), unreliable visual fields, signal strength of less than 15 on Heidelberg Spectralis OCT, use of ocular medication that may affect pupillary reaction like miotics or mydriatics, any laser procedure or intravitreal injections for any ocular disease, and intraocular surgeries (except uncomplicated cataract surgery that is older than six months) were excluded. An a priori power analysis was performed using G*Power version 3.1. Assuming a medium effect size (f² = 0.15), a significance level of 0.05, a statistical power of 90%, and three predictor variables (inter-eye RNFL difference, MD difference, and VFI difference), the minimum required sample size was calculated to be 100 participants. To allow for potential missing data and to ensure stable parameter estimation in the planned ordinal logistic regression analysis, a target sample size of 120 participants was adopted.

The eligible participants were recruited and informed written consent were taken either from them or parents/guardian if less than 18 years old. Each participant underwent comprehensive ocular examination that included best corrected visual acuity (BCVA) using Snellen’s chart, intraocular pressure (IOP) using Goldmann applanation tonometer, anterior segment and posterior segment examination using 78 Diopter condensing lens, pupil reaction and gonioscopy. RAPD grading was performed independently by two trained third-year ophthalmology residents using a standardized swinging flashlight test protocol under supervision of glaucoma consultant. Prior to data collection, both examiners underwent a calibration session to ensure uniform application of grading criteria. In cases of discrepancy, final grading was determined by consensus after joint re-evaluation and consensus under consultant supervision. The pupillary examination was performed with the lights in the room turned off.

The participants were given 60 seconds to adapt to the light conditions before commencing the test. A bright, portable light source (an indirect ophthalmoscope) was held around 30 cm from the participants’ eyes and held constant for three seconds while they were instructed to focus on a far-off object (6 meters away) to measure their pupillary reaction. The light source was then quickly flashed into the opposite eye and maintained there for three more seconds. Clinical Plus grading was then used to record RAPD, as explained below; Grade 1+: early re-dilatation after initial weak constriction; Grade 2+: no initial stalling or constriction followed by dilatation; Grade 3+: instant dilatation; and Grade 4+: fixed amaurotic pupil or non-reactive pupil in the affected eye. Visual fields were assessed using the Humphrey Field Analyzer 3 (HFA-3; Carl Zeiss Meditec, Dublin, CA, USA), employing the 24-2 Swedish Interactive Threshold Algorithm (SITA)-Standard strategy. Only reliable fields (fixation losses <20%, false positives/negatives <15%) were included.

The following visual field parameters were recorded; mean deviation (MD), visual field index (VFI), and the inter-eye differences in MD and VFI. These differences were calculated by subtracting visual field parameters of the worse (RAPD positive) eye from those of the better (RAPD negative) eye. The percentage decrease for these parameters were also calculated and noted. The retinal nerve fiber layer (RNFL) thickness around the optic disc was assessed by using Heidelberg Spectralis OCT. About three circular scans, each 3.4 mm in diameter, centered on the optic disc were taken after pupillary dilatation, which was averaged by the Fast RNFL thickness program. The RNFL thickness was recorded as following: RNFL-Global, Superior, Inferior, Nasal, and Temporal quadrants. Inter-eye RNFL thickness were calculated by subtracting RNFL thickness of worse eye from those of better eye.^14^

The percentage decrease for these parameters were also calculated and noted. To maintain accuracy all scans were required to have a quality of 15 or higher. All the data were recorded on a predesigned proforma. The Snellen’s VA was converted to LogMAR for data analysis. Data were analyzed using IBM SPSS Statistics for Windows, version 24 (IBM Corp., Armonk, N.Y., USA). Mean ± SD for continuous variables (age, BCVA, SE, CDR, IOP, maximum IOP (Tmax), RNFL thicknesses, MD, and VFI) and frequency for categorical variables (gender, worse eye, RAPD grades, diagnosis, and stage of glaucoma) were calculated. Hodapp-Parrish-Anderson criteria was used for glaucoma staging.^15^ Ordinal logistic regression was performed to assess the relationship between the grades of RAPD and the inter-eye differences in RNFL thickness, MD, and VFI. A p value of less than 0.05 was set for statistical significance.

## RESULTS

A total of 467 participants met the inclusion criteria of which 341 were excluded due to various reasons (such as low vision: 124, trabeculectomy: 110, outside age limits: 20, YAG peripheral iridotomy: 14, high myope: 09, unreliable visual fields: 08, recent cataract surgery: 07, retinal detachment: 07, uveitis: 07, pars plana vitrectomy: 07, trauma: 06, retinal vein occlusion: 04, selective laser trabeculoplasty: 03, corneal haze: 03, segmentation errors on OCT: 03, macular edema: 02, epiretinal membrane: 02, ischemic optic neuropathy: 02, panretinal photocoagulation: 01, vitreous hemorrhage: 01, and full thickness macular hole: 01). The final cohort consisted of 126 individuals, with a mean age of 52.48 ± 14.63 years, predominantly males (77%). Primary open angle glaucoma was the most prevalent diagnosis, with 38 (30.2%) of better eyes and 109 (86.5%) of worse eyes presented with advanced stages. Table-I highlights the baseline demographics of study participants. Table-II compares the baseline clinical profile of the better and worse eye which were all statistically significant (p<0.05). Tables-III and IV show analyses of OCT and VF parameters with subgroup analysis according to RAPD grades. The inter-eye differences among all the parameters of OCT-RNFL whether global or sectoral and of VF whether MD or VFI were statistically significant (p<0.001).

**Table-I:** Baseline demographics of study participants.

*Characteristics N=126*		*Frequency (%)*
Age, mean (SD) in years	52.48 (14.63)	
Gender, n (%)	Male	97 (77)
	Female	29 (23)
Worse eye, n (%)	Right	62 (49.2)
	Left	64 (50.8)
Lens status of better eye, n (%)	Phakic	109 (86.5)
	Pseudophakic	17 (13.5)
Lens status of worse eye, n (%)	Phakic	99 (78.6)
	Pseudophakic	27 (21.4)
Diagnosis of better eye, n (%)	Non-glaucomatous	26 (20.6)
	POAG	55 (43.7)
	SIG	12 (9.5)
	OHT	10 (7.9)
	PXF-G	9 (7.1)
	NTG	5 (4)
	PACG	4 (3.2)
	JOAG	2 (1.6)
	PACS	2 (1.6)
	PAC	1 (0.8)
Diagnosis of worse eye, n (%)	POAG	73 (57.9)
	PXF-G	22 (17.5)
	SIG	16 (12.7)
	PACG	7 (5.6)
	NTG	6 (4.8)
	JOAG	2 (1.6)
Stage of better eye, n (%)	Not stageable	39 (31)
	Early	15 (11.9)
	Moderate	34 (27)
	Advanced	38 (30.2)
Stage of worse eye, n (%)	Early	2 (1.6)
	Moderate	15 (11.9)
	Advanced	109 (86.5)
RAPD grades, n (%)	Grade 1	84 (66.7)
	Grade 2	29 (23)
	Grade 3	12 (9.5)
	Grade 4	1 (0.8)

N = total sample size, n = frequency, % = percentage, SD = standard deviation, POAG = primary open angle glaucoma, SIG = steroid induced glaucoma, OHT = ocular hypertension, PXF-G = pseudoexfoliation glaucoma, NTG = normal tension glaucoma, PACG = primary angle closure glaucoma, JOAG = juvenile open angle glaucoma, PACS = primary angle closure suspect, PAC = primary angle closure, RAPD = relative afferent pupillary defect.

**Table-II:** Baseline comparison in demographics of clinical profile of study participants.

Characteristics	Better eye (mean ± SD)	Worse eye (mean ± SD)	p-value*
VA (LogMAR)	0.34 ± 0.34	0.6 ± 0.5	<0.001
BCVA (LogMAR)	0.13 ± 0.22	0.34 ± 0.48	<0.001
Refractive error (SE)	-0.2 ± 1.26	-0.51 ± 1.6	0.016
IOP (mmHg)	16.68 ± 5.68	19.59 ± 8.19	<0.001
Tmax (mmHg)	23.72 ± 9.08	31.7 ± 12.35	<0.001
CDR	0.61 ± 0.26	0.91 ± 0.09	<0.001

SD = standard deviation, VA = visual acuity, LogMAR = logarithm of minimum angle of resolution, BCVA = best corrected visual acuity, SE = spherical equivalent, IOP = intraocular pressure, mmHg = millimeter of mercury, Tmax = maximum intraocular pressure, CDR = cup-to-disc ratio.

* Paired sample t-test was applied.

**Table-III:** OCT parameter analysis with subgroup analysis according to RAPD grades.

Parameters (N=126)		Better eye (mean ± SD)	Worse eye (mean ± SD)	p-value*	Inter-eye difference (mean ± SD)	Percentage difference (mean ± SD)
RNFL thickness Global (microns)	All cases	79.8 ± 21.12	50.3 ± 15.49	<0.001	29.45 ± 20.27	34.39 ± 19.82
	Grade 1	80.35 ± 21.10	51.89 ± 16.11	<0.001	28.38 ± 21.21	32.82 ± 20.73
	Grade 2	78.76 ± 19.70	49.34 ± 13.75	<0.001	29.41 ± 16.86	35.30 ± 16.54
	Grade 3	77.08 ± 26.09	41.75 ± 13.47	<0.001	35.33 ± 21.62	41.80 ± 20.43
	Grade 4	97	47	NA	50.00	51.5
RNFL thickness Superior (microns)	All cases	96.04 ± 29.15	59.29 ± 20.56	<0.001	36.74 ± 29.55	37.34 ± 22.17
	Grade 1	97.04 ± 29.41	62.08 ± 22.17	<0.001	34.94 ± 30.86	36.63 ± 23.17
	Grade 2	93.97 ± 26.89	56.52 ± 16.841	<0.001	37.45 ± 25.65	36.33 ± 20.10
	Grade 3	92.33 ± 34.90	46.17 ± 10.19	<0.001	46.17 ± 30.17	44.02± 21.05
	Grade 4	117	63	NA	54	46.10
RNFL thickness Inferior (microns)	All cases	100.06 ± 33.05	57.1 ± 21.32	<0.001	42.95 ± 33.3	40 ± 21.47
	Grade 1	100.51 ± 32.49	59.26 ± 22.52	<0.001	41.25 ± 34.83	38.33 ± 22.35
	Grade 2	100.38 ± 32.41	55.90 ± 19.36	<0.001	44.48 ± 28.06	42.14 ± 18.39
	Grade 3	94.33 ± 41.16	45.83 ± 14.03	0.001	48.50 ± 35.81	44.69 ± 22.47
	Grade 4	121	46	NA	75	61.90
RNFL thickness Nasal (microns)	All cases	62.78 ± 19.42	41.64 ± 16.2	<0.001	21.13 ± 19.67	33.29 ± 21.72
	Grade 1	63.12 ± 20.65	42.95 ± 16.13	<0.001	20.17 ± 19.89	30.74 ± 21.45
	Grade 2	63.03 ± 15.45	39.55 ± 16.52	<0.001	23.48 ± 17.10	38.04 ± 22.63
	Grade 3	59 ± 20.90	36.75 ± 16.28	0.011	22.17 ± 25.42	40.05 ± 20.68
	Grade 4	72	51	NA	21	29.10
RNFL thickness Temporal (microns)	All cases	60.16 ± 14.75	43.85 ± 14.31	<0.001	16.35 ± 17.46	28.37 ± 20.16
	Grade 1	60.68 ± 14.18	44.24 ± 14.27	<0.001	16.49 ± 17.19	28.79 ± 19.49
	Grade 2	57.07 ± 14.3	45.45 ± 12.52	0.001	11.66 ± 16.04	21.91 ± 17.62
	Grade 3	62.58 ± 19.35	38.33 ± 18.29	0.001	24.25 ± 19.29	38.49 ± 25.44
	Grade 4	77	31	NA	46	59.70

OCT = optical coherence tomography, VF = visual field, N = total sample size, SD = standard deviation, RNFL = retinal nerve fiber layer, dB = decibel, % = percentage, NA = not applicable.

* Paired sample t-test was applied.

The greatest inter-eye and percentage differences for RNFL-G, RNFL-I, RNFL-T, and MD were found in grade 3 RAPD while RNFL-N and VFI showed the greatest inter-eye difference in RAPD of grade 2. Fig.1A–1C illustrate the relationship between inter-eye differences in RNFL thickness, MD, and VFI with the RAPD grade. Each box plot represents the median, interquartile range, and outlier values for the respective variables. Fig.1A demonstrates that greater inter-eye differences in RNFL thickness are associated with higher RAPD grades, indicating a progressive structural asymmetry in eyes with severe glaucomatous damage. Fig.1B shows that inter-eye MD differences similarly increase with RAPD grade, suggesting a direct correlation between functional visual field loss and RAPD severity. Fig.1C illustrates that inter-eye differences in VFI also follow a comparable trend, with larger discrepancies corresponding to higher RAPD grades, reinforcing the relationship between visual function loss and pupillary response asymmetry.

**Fig.1A:**
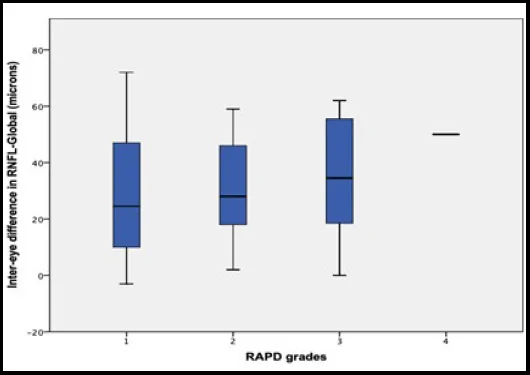
Box plots showing the median, interquartile range and outside values of inter-eye difference in RNFL-G thickness and the RAPD grades.

**Fig.1B:**
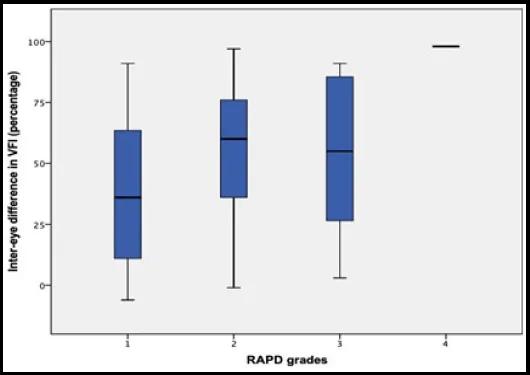
Box plots showing the median, interquartile range and outside values of inter-eye difference in VFI and the RAPD grades.

**Fig.1C:**
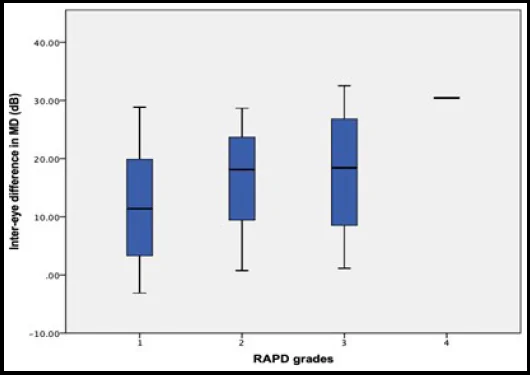
Box plots showing the median, interquartile range and outside values of inter-eye difference in MD and the RAPD grades.

We performed ordinal regression to assess whether differences in RNFL thickness, mean deviation (MD), and visual field index (VFI) between the two eyes predict RAPD grades (1 to 4). The analysis showed that MD difference was a significant predictor of RAPD grade (p = 0.040). For every 1 dB increase in MD difference, the odds of having a higher RAPD grade increased by 22.5% (odds ratio = 1.225). In contrast, RNFL thickness differences (global and sectoral) and VFI differences were not significantly associated with RAPD grade (all p>0.05) as shown in Table-V. The association between the glaucoma stage of the worse eye and RAPD grades were sought which were found statistically nonsignificant (p=0.345) as shown in Table-VI.

**Table-IV:** VF parameter analysis with subgroup analysis according to RAPD grades.

Parameters (N=126)		Better eye (mean ± SD)	Worse eye (mean ± SD)	p-value*	Inter-eye difference (mean ± SD)	Percentage difference (mean ± SD)
Mean deviation (dB)	All cases	-7.9 ± 7.1	-21.46 ± 9.7	<0.001	13.54 ± 9.28	
	Grade 1	-7.33 ± 6.76	-19.18 ± 9.73	<0.001	11.83 ± 8.75	
	Grade 2	-7.72 ± 7.62	-24.04 ± 8.46	<0.001	16.32 ± 8.84	
	Grade 3	-12.78 ± 12.26	-30.12 ± 4.4	<0.001	17.35 ± 10.83	
	Grade 4	-3.06	-33.49	NA	30.43	
Visual field index (%)	All cases	83.05 ± 23.36	38.7 ± 32.54	<0.001	44.33 ± 30.12	59.32 ± 54.63
	Grade 1	84.90 ± 19.88	45.48 ± 33.03	<0.001	39.42 ± 29.12	53.64 ± 63.31
	Grade 2	84.79 ± 22.41	31.62 ± 29.02	<0.001	53.14 ± 28.39	65.10 ± 29.46
	Grade 3	64.50 ± 38.81	11.50 ± 15.52	<0.001	53 ± 33.51	81.75 ± 20.77
	Grade 4	99	1	NA	98	98.90

VF = visual field, N = total sample size, SD = standard deviation, RNFL = retinal nerve fiber layer, dB = decibel, % = percentage, NA = not applicable.

* Paired sample t-test was applied.

**Table -V:** Ordinal Logistic Regression of structural and functional predictors of Relative Afferent Pupillary Defect severity.

Predictor (N=126)	Unadjusted				Adjusted			
	B	OR (Exp(B))	95% CI	p-value	B	OR (Exp(B))	95% CI	p-value
Inter-eye difference in RNFL-Global	0.010	1.010	0.992–1.028	0.284	–0.022	0.978	0.868–1.102	0.714
Inter-eye difference in RNFL-Superior	0.007	1.007	0.995–1.020	0.249	0.002	1.002	0.964–1.042	0.924
Inter-eye difference in RNFL-Inferior	0.005	1.005	0.994–1.016	0.344	0.002	1.002	0.968–1.037	0.916
Inter-eye difference in RNFL-Nasal	0.007	1.007	0.989–1.025	0.450	0.000	1.000	0.965–1.038	0.979
Inter-eye difference in RNFL-Temporal	0.004	1.004	0.983–1.025	0.712	0.001	1.001	0.965–1.039	0.978
Inter-eye difference in MD	0.066	1.068	1.024–1.114	0.002	0.203	1.225	1.009–1.489	0.040
Inter-eye difference in VFI	0.017	1.017	1.005–1.030	0.007	–0.036	0.965	0.910–1.021	0.218

N = total sample size, B = beta coefficient, OR = odds ratio, CI = confidence interval, RNFL = retinal nerve fiber layer, MD = mean deviation, VFI = visual field index.

**Table-VI:** Association of RAPD grades with glaucoma staging of worse eye.

Characteristics (N=125)		RAPD Grade			p-value*
		1	2	3	
Glaucoma Stage, n (%)	Early	2 (100)	0 (0)	0 (0)	0.345
	Moderate	13 (86.7)	2 (13.3)	0 (0)	
	Advanced	69 (63.3)	27 (24.8)	12 (11)	
Total		84 (66.7)	29 (23)	12 (9.5)	125 (100)

N = total sample size, n = frequency, % = percentage, RAPD = relative afferent pupillary defect. RAPD Grade 4 (n=1) was excluded.

* chi-square test was performed (when the expected count was < 5 in 25% of the cells then the Fisher exact test was applied).

## DISCUSSION

The study aimed to investigate the relationship between visual field indices and RNFL thickness with RAPD in asymmetric glaucoma. It found significant associations between RAPD grades and inter-eye differences in VFI, MD, and RNFL thickness. In our cohort, clinically detectable RAPD was observed when the inter-eye differences were approximately 32.8% in RNFL thickness, 11.8 dB in MD, and 53.6% in VFI. These values should be interpreted as empirical thresholds observed in this study rather than universal biological cut-off values. The strongest link to RAPD severity was found in inter-eye asymmetry in MD, while no significant correlation was noted between RAPD grade and glaucoma stage.

Our research found significant variations in RNFL thickness, MD, and VFI across all grades of RAPD, with the most pronounced differences in grade 3, except for RNFL in the nasal sector. This aligns with prior studies indicating similar inter-eye discrepancies. Our focus on glaucoma patients distinguished our study from previous mixed-population analyses.^1^,^2^,^5^,^6^,^10^,^11^,^16^,^17^ While previous researches noted a 17–27% reduction in RNFL thickness necessary for RAPD detection,^1^,^5^,^6^,^17^,^18^ we reported a higher threshold of 32.8%, potentially due to demographic and disease variability, as well as dissimilar RAPD assessment methods. Most importantly, our study concentrated on glaucoma patients only, in contrast to earlier research that included a mixed population. This addressed Aradhna Mishra et al.’s suggestion for disease-specific studies.^2^ Additionally, functional parameters indicated larger reductions in MD and VFI for detectable RAPD, with an inter-eye MD difference of at least 11.8 dB, aligning with previous findings that suggested a difference of 6.17 to 10.15 dB.^10^,^16^ Although a Thai study found that VFI was a greater indicator of RAPD,^10^ it only included 15.4% of glaucoma cases, which limited direct comparability.

Our findings also showed that RAPD severity was better predicted by inter-eye MD differences than by VFI asymmetry or RNFL thickness. Each 1 dB increase in the inter-eye MD difference was associated with a 22.5% increase in the odds of belonging to a higher RAPD category (OR = 1.225). Although inter-eye MD difference was significantly associated with RAPD severity, the effect estimate should be interpreted as a statistical association within the proportional odds model rather than as evidence that each 1 dB change produces an equivalent biological or functional impact. This distinction is important because the relationship between MD and glaucomatous functional loss is known to be non-linear, particularly in advanced disease. This result emphasizes how much more sensitive MD is than VFI at identifying functional asymmetry in glaucoma. MD represents entire visual field depression, comprising both central and peripheral deficiencies, in contrast to VFI, which focusses on central vision and stays mostly stable throughout early or peripheral field loss.^19^ Since generalized afferent pathway impairment is reflected in RAPD, MD seems to be more appropriate for identifying the asymmetry that affects RAPD grading. While our study demonstrated a stronger link between MD difference and RAPD severity, other study has suggested VFI as a better predictor.^10^

This apparent difference most likely results from different study populations: previous research included a variety of RAPD etiologies (such as compressive lesions, ischemic optic neuropathy, and optic neuritis), where central vision is frequently primarily impacted, increasing the sensitivity of VFI. On the other hand, MD was the more dependable parameter since we only looked at asymmetric glaucomatous optic neuropathy, which is characterized by diffuse field loss. Interestingly, in the same study,^10^ glaucoma patients with severe RAPD demonstrated preserved VFI values despite having significantly reduced MD, suggesting that VFI may underestimate the extent of visual field loss in cases with dense, localized defects. Their observation further supports our findings that inter-eye difference in MD seems to be better predictor of RAPD than VFI. Remarkably, in our study, RNFL thickness variations between eyes did not significantly predict the severity of RAPD. The presence of advanced glaucoma cases (about 30% of better eyes) may be an explanation of this, as the optical coherence tomography (OCT) floor effect causes RNFL measurements to plateau. In a comparable manner, Sarezky et al. also proposed that in advanced glaucoma, RNFL asymmetry loses some of its ability to predict for RAPD.^20^ Similarly, prior research also support our findings that OCT may have reduced sensitivity in late-stage disease and that RAPD examination may be less helpful when disease asymmetry is small or both eyes are significantly affected.^21^,^22^

The study examined the correlation between RAPD severity and glaucoma stage, noting a non-significant trend of increasing RAPD grades with disease advancement (p = 0.345). Most advanced glaucoma cases exhibited higher RAPD grades, while early and moderate glaucoma typically showed grade 1 RAPD. Limitations such as a small number of grade 4 RAPD cases restricted the ability to find meaningful correlations. The findings suggest that RAPD grading reflects inter-eye asymmetry rather than overall disease stage, underscoring the importance of combining functional and structural evaluations in glaucoma assessments. Incorporating RAPD evaluations into clinical practice can aid in monitoring disease progression and identifying at-risk patients. RAPD may serve as a simple clinical marker of inter-eye structural and functional asymmetry in glaucoma, reflecting ganglion cell dysfunction across a broader disease spectrum rather than being confined to end-stage disease. There are various benefits of incorporating RAPD evaluation into clinical practice. It can assist in tracking the course of the disease and may help identify patients who have severe inter-eye asymmetry and are more likely to lose vision. To maintain binocular vision, immediate action may be necessary if changes in the RAPD grade over time reveal progressive optic nerve damage in the less affected eye.

### Strengths:

Strengths of the study include a larger sample size and a focus on asymmetric glaucoma which has addressed the concerns of previous studies.

### Limitations:

limitations involve limited generalizability and under-representation of early glaucoma cases.

## CONCLUSION

The best predictor of RAPD severity among the metrics examined was inter-eye asymmetry in MD. Importantly, RAPD grading seems to represent ocular asymmetry rather than glaucoma stage, confirming its use as a diagnostic tool for clinical evaluation and tracking and as a marker of glaucomatous optic nerve dysfunction.

### Future directions:

Future research should build on these findings by evaluating larger and more diverse patient cohorts to confirm the generalizability of MD asymmetry as an independent predictor of RAPD severity. Longitudinal studies are also needed to determine whether intereye MD differences can predict subsequent RAPD progression or functional decline. In addition, earlier stages of glaucoma warrant focused investigation, as subtle structural or functional asymmetries may be more informative before advanced damage occurs. Incorporating newer imaging modalities—such as macular GCIPL asymmetry, OCTA perfusion metrics, and automated RAPD quantification—may further clarify structure–function relationships and help refine the clinical utility of RAPD assessment in glaucoma care. Future research using AI-based RAPD quantification may enhance accuracy and clinical utility.

### AI tool(s) Statement:

During the preparation of this work the author(s) used ChatGPT and Quillbot for coherence and paraphrasing. After using this tool/service, the author(s) reviewed and edited the content as needed and take(s) full responsibility for the content of the publication.

### Authors’ Contribution:

**YJM:** Contributed to the concept, design, literature search, data analysis, statistical analysis, manuscript preparation, manuscript editing,

**ARK, AK, HA, SF, RZ:** Contributed to the design, literature search, data acquisition, manuscript preparation, manuscript editing,

All authors have read and approved the final version of manuscript. They are also accountable for the integrity of the study.
